# Cardiorenal Safety of OTC Analgesics

**DOI:** 10.1177/1074248417751070

**Published:** 2018-02-08

**Authors:** William B. White, Robert A. Kloner, Dominick J. Angiolillo, Michael H. Davidson

**Affiliations:** 1 Calhoun Cardiology Center, University of Connecticut School of Medicine, Farmington, CT, USA; 2 HMRI Cardiovascular Research Institute, Huntington Medical Research Institutes, Pasadena, CA, USA; 3 Cardiovascular Division, Department of Medicine, Keck School of Medicine, University of Southern California, Los Angeles, CA, USA; 4 Division of Cardiology, University of Florida College of Medicine, Jacksonville, FL, USA; 5 Preventive Cardiology, The University of Chicago Medicine, Chicago, IL, USA

**Keywords:** nonsteroidal anti-inflammatory drugs, acetaminophen, coxibs, pain, over-the-counter, safety

## Abstract

Over-the-counter analgesics are used globally for the relief of acute pain. Although effective, these agents can be associated with adverse effects that may limit their use in some people. In the early 2000s, observations from clinical trials of prescription-strength and supratherapeutic doses of nonselective and cyclooxygenase-2-selective nonsteroidal anti-inflammatory drugs (NSAIDs) raised safety concerns regarding the risk of cardiovascular adverse effects with the use of these medications. Subsequently, the US Food and Drug Administration mandated additional study of the cardiovascular safety of NSAIDs for a more comprehensive understanding of their risk. As these data were being collected, and based on a comprehensive review of prescription data and the recommendations of the US Food and Drug Administration Advisory Committee, the warning labels of over-the-counter NSAIDs were updated to emphasize the potential cardiovascular risks of these agents. The recently reported “Prospective Randomized Evaluation of Celecoxib Integrated Safety versus Ibuprofen or Naproxen” (PRECISION) trial, in which participants with osteoarthritis or rheumatoid arthritis and underlying cardiovascular risk factors were treated with prescription-strength celecoxib, ibuprofen, or naproxen, revealed similar rates of cardiovascular events (death from cardiovascular causes including hemorrhagic death, nonfatal myocardial infarction, or nonfatal stroke) among the 3 treatment groups. Although informative, the cardiovascular safety findings derived from PRECISION cannot be extrapolated to the safety of the over-the-counter pain relievers ibuprofen and naproxen, given that the doses used were higher (mean [standard deviation]: ibuprofen, 2045 [246] mg; naproxen, 852 [103] mg) and the durations of use longer (∼20 months) than recommended with over-the-counter use of NSAIDs, which for ibuprofen is up to 10 days. This review discusses the cardiorenal safety of the most commonly used over-the-counter analgesics, ibuprofen, naproxen, and acetaminophen. Available data suggest that there is little cardiovascular risk when over-the-counter formulations of these agents are used as directed in their labels.

## Introduction

Pain is a common problem, and over-the-counter (OTC) analgesic agents alleviate pain in populations around the globe. Among the most commonly used OTC analgesic agents are nonsteroidal anti-inflammatory drugs (NSAIDs) and acetaminophen (paracetamol; APAP; Table 1).^1-15^

**Table 1.** Commonly Available Analgesics.^a^

Product	Status (ie, Rx, OTC, or Both)	Common Brands	OTC Indication(s)	OTC Dose and Duration	Rx Indication(s)	Rx Dose
Acetaminophen^1,2^	Both Rx (in combination with another analgesic, usually an opioid) and OTC	Tylenol; opioid combinations include Vicodin, Percocet, Lortab	Temporarily relieves minor aches and pains due to the common cold, headache, backache, minor pain of arthritis, toothache, premenstrual and menstrual cramps; also temporarily reduces fever	Adults and children ≥12 years of age: take 2 caplets (650-1000 mg) every 4-6 hours while symptoms last; do not take more than 10 caplets in 24 hours; do not use for more than 10 days unless directed by a doctor	Acetaminophen/opioid combinations are indicated for moderate to severe pain	Variable
				Children 6-11 years of age: take 1 caplet every 4-6 hours while symptoms last; do not take more than 5 caplets in 24 hours; do not use for more than 5 days unless directed by a doctor		
Diclofenac^3-5^	Both Rx and OTC	Arthrotec, Cataflam, Voltaren, Cambia	Treatment of temporary mild to moderate pain	12.5-25 mg 3 times a day (maximum daily dose is 75 mg/d)	OA RA AS Dysmenorrhea Mild to moderate pain Migraine headache	1) and 2) 50 mg PO every 8-12 hours 3) 25 mg PO 4-5 times/d 4) and 5) 100 mg PO once; then 50 mg every 8 hours 6) 50 mg (once)
Ibuprofen^4,6-8^	Both Rx and OTC	Advil, Motrin	Minor aches and pains due to headache, muscle aches, backache, minor pain of arthritis, toothache, menstrual cramps, and the common cold; also temporarily reduces fever	In adults and children 12 years of age and older: 200 mg every 4-6 hours while symptoms persist; if pain or fever does not respond to 200 mg, then 400 mg may be used; do not exceed 6 tablets (1200 mg) in 24 hours. Stop use if pain worsens or lasts more than 10 days or if fever lasts more than 3 days	OA RA Mild to moderate pain Primary dysmenorrhea	1) and 2) 1200-3200 mg daily (300 mg 4 times a day; 400 mg, 600 mg, or 800 mg 3 times a day or 4 times a day) 3) 400 mg every 4-6 hours as needed 4) 400 mg every 4 hours as needed
Indomethacin^4,9^	Rx	Indocin, Indocin SR	N/A	N/A	Moderate to severe RA, including acute flares Moderate to severe AS Moderate to severe OA Acute painful shoulder (bursitis and/or tendinitis) Acute gouty arthritis	1), 2), and 3) total daily dose of 150-200 mg 4) 75-150 mg daily in 3 or 4 divided doses 5) 50 mg 3 times daily
Ketoprofen^4,10^	Rx	Actron, Orudis, Orudis KT, Orudis SR, Oruvail	N/A	N/A	OA RA	1) and 2) 100-200 mg daily in divided dosage (2-4 times daily); SR: 100-200 mg once daily
Meloxicam^4,11^	Rx	Mobic	N/A	N/A	OA RA JRA	1) and 2) 7.5-15 mg/d 3) 7.5 mg/d in children ≥60 kg, should not be used in children <60 kg
Naproxen, naproxen sodium^4,12^^,^^13,b^	Both Rx and OTC	Aleve, Naprosyn, Anaprox, Anaprox DS, EC-Naprosyn	Minor aches and pains due to minor pain of arthritis, muscle aches, backache, menstrual cramps, headache, toothache, and the common cold; also temporarily reduces fever	220 mg (sodium salt), NTE 3 tablets (600 mg naproxen equivalents) in 24 hours; stop use if pain worsens or lasts more than 10 days or if fever worsens or lasts more than 3 days	OA RA AS Polyarticular juvenile idiopathic arthritis Management of pain, primary dysmenorrhea, acute tendonitis, and bursitis Acute gout	1), 2), and 3) Naprosyn 250-500 mg twice a day; Anaprox-DS 275-550 mg twice a day; EC-Naprosyn 375-500 mg twice a day 4) 10 mg/kg given in 2 divided doses 5) Naprosyn 500 mg initially, then 250 mg every 6-8 hours (NTE 1250 mg) 6) Anaprox DS 825 mg initially, then 275 mg every 8 hours
Piroxicam^4,14^	Rx	Feldene	N/A	N/A	OA RA	20 mg once daily
Celecoxib^4,15^	Rx	Celebrex	N/A	N/A	OA RA JRA AS Acute pain and primary dysmenorrhea	200 mg/d or 100 mg twice a day 100-200 mg twice a day ≥10 kg to ≤ 25 kg: 50 mg twice a day; >25 kg: 100 mg twice a day 200 mg/d or 100 mg twice a day; may increase to 400 mg/d 400 mg initially, followed by 200 mg if needed on first day; subsequently 200 mg twice a day as needed

Abbreviations: AS, ankylosing spondylitis; JRA, juvenile rheumatoid arthritis; N/A, not applicable; NTE, not to exceed; OA, osteoarthritis; OTC, over-the-counter; PO, by mouth; RA, rheumatoid arthritis; Rx, prescription.

^a^Brand names are the trademarks of their respective owners.

^b^220 mg of naproxen sodium contains 200 mg of naproxen.

Large-scale population studies confirm that OTC analgesic use is common around the world.^16-19^ In the Nurses’ Health Study (NHS) I survey (N = 86 985), 22% of US nurses aged 52 to 77 years reported use of APAP and 27% reported use of an NSAID at least once per week.^20^ Among US nurses aged 33 to 51 years who participated in the NHS II survey (N = 93 002), 25% reported using APAP and 42% reported using an NSAID at least once per week. In a 2005 Scottish postal survey of 2708 individuals ≥18 years, 37% of respondents reported OTC analgesic use (ibuprofen, APAP, aspirin [ASA], or compound preparations) over the preceding 2 weeks.^18^ In Norway, a survey of individuals ≥20 years conducted between 2006 and 2008 found that 47% of the 39 767 respondents had used OTC analgesics (APAP, NSAIDs, ASA) at least once per week over the last month.^16^

Beginning in 2000,^21^ an increased cardiovascular (CV) event risk was noted within some patient populations during clinical trials of cyclooxygenase (COX)-2-selective NSAIDs^22,23^ and separately in a meta-analysis that included nonselective NSAIDs.^24^ In a 2005 long-term, placebo-controlled clinical trial evaluating the potential of the standard dose of the COX-2-selective NSAID rofecoxib (25 mg daily) to prevent adenoma recurrence in participants with a history of colorectal adenomas (the Adenomatous Polyp Prevention on Vioxx Trial), an increased incidence of serious CV adverse events (AEs), particularly myocardial infarction (MI) and stroke, was observed in participants receiving rofecoxib compared with participants receiving placebo.^22^ Subsequently, the trial was suspended, and in 2004, rofecoxib was withdrawn from the market.^22,25^ Thereafter, the Adenoma Prevention with Celecoxib study also found an increased risk of serious CV events versus placebo with a supratherapeutic dose of celecoxib (800 mg daily).^23^ The data and safety monitoring board recommended study termination,^23^ but celecoxib 200 mg capsules remained on the market. A 2006 meta-analysis comparing serious vascular events between COX-2 inhibitors and placebo or nonselective NSAIDs showed that some traditional NSAIDs might also be associated with atherothrombotic AEs.^24^

Growing concerns related to the data detailed above resulted in the conduct of a US Food and Drug Administration (FDA)-mandated CV safety risk study entitled “Prospective Randomized Evaluation of Celecoxib Integrated Safety versus Ibuprofen or Naproxen” (PRECISION), comparing prescription-strength celecoxib, naproxen, and ibuprofen.^26^ Results of that trial, detailing CV, gastrointestinal (GI), and renal safety outcomes in participants aged ≥18 years with increased CV risk who required daily NSAID treatment due to pain from osteoarthritis or rheumatoid arthritis, were published in 2016.^26^ More than 24 000 participants were randomized to celecoxib (mean [SD] daily dose, 209 [37] mg), ibuprofen (2045 [246] mg), or naproxen (852 [103] mg); treatment was maintained for an average of 20 months. Approximately two-thirds of the participants stopped study treatment before the end of the trial, and numerous sensitivity analyses were therefore performed for the primary and secondary end points. The first occurrence of an AE meeting the Antiplatelet Trialists Collaboration (APTC) criteria (ie, death from CV causes including hemorrhagic death, nonfatal MI, or nonfatal stroke) was the primary safety outcome of interest. At the end of the study, similar rates of AEs meeting APTC criteria were noted with each treatment (celecoxib: 2.3%, ibuprofen: 2.7%, and naproxen: 2.5%). Celecoxib was noninferior to ibuprofen, with a hazard ratio (HR) of 0.85 (95% confidence interval [CI]: 0.70-1.04; *P* < .001) and noninferior to naproxen, with an HR of 0.93 (95% CI: 0.76-1.13; *P* < .001). On-drug sensitivity analyses produced results that were comparable to and supportive of the intention-to-treat analysis. The authors concluded that the doses of celecoxib evaluated were associated with CV risk similar to that with nonselective NSAIDs.^26^ In contrast to those findings, celecoxib was associated with a significantly lower risk of GI AEs than naproxen (*P* = .01) or ibuprofen (*P* = .002) and with a significantly lower risk of renal AEs versus ibuprofen (*P* = .004), but a similar renal AE risk compared with naproxen (*P* = .19).

The PRECISION safety findings for ibuprofen and naproxen cannot be extrapolated to OTC dosing, however. Recommended OTC doses are lower than prescription doses and are designated for much shorter use durations.^27,28^ Further, participants in the PRECISION study were at an increased risk of CV events and as such may not represent the typical OTC analgesic consumer. The safety of OTC analgesics remains relevant, however, given the high frequency of OTC NSAID use.

To date, most published trials reporting the safety of OTC NSAIDs extrapolate data from “prescription” NSAIDs used at “low doses,” but these studies typically involve use over an extended period that is considerably longer than recommended for OTC use.^29-36^ In this article, we review the CV safety of commonly used analgesics (ibuprofen, naproxen, and APAP) when administered in doses consistent with OTC use.

## Cardiovascular Pharmacology of COX Inhibition

### Cyclooxygenase 1 and Cyclooxygenase 2

Both COX-1 and COX-2 are isoforms of the cyclooxygenase enzyme mediating the conversion of arachidonic acid to prostaglandin H_2_, the substrate for 5 different prostanoids, PGD_2_, PGE_2_, PGF_2α_, prostacyclin (PGI_2_), and thromboxane A_2_.^37,38^ Cyclooxygenase 1 is constitutively active and is expressed in most tissues, including kidney, lung, stomach, duodenum, jejunum, ileum, colon, and cecum. It functions in gastric cytoprotection, vascular homeostasis, platelet aggregation, and maintenance of normal kidney function.^37-41^ Cyclooxygenase 2 is an inducible enzyme expressed in the brain, kidney, and possibly in the female reproductive system.^37,42,43^ Cyclooxygenase 2 expression is increased during states of inflammation.^44^ Cyclooxygenase 2 is a key source of PGI_2_ and is cardioprotective in ischemia–reperfusion injury.^45,46^ Hence, selective COX-2 inhibition could lead to decreased antithrombotic PGI_2_, enhanced leukotriene synthesis, and increased reactive oxygen species and consumption of antithrombotic nitric oxide.^47^

### Cyclooxygenase Selectivity and Potential CV Ramifications of COX Inhibition

**Figure 1.**
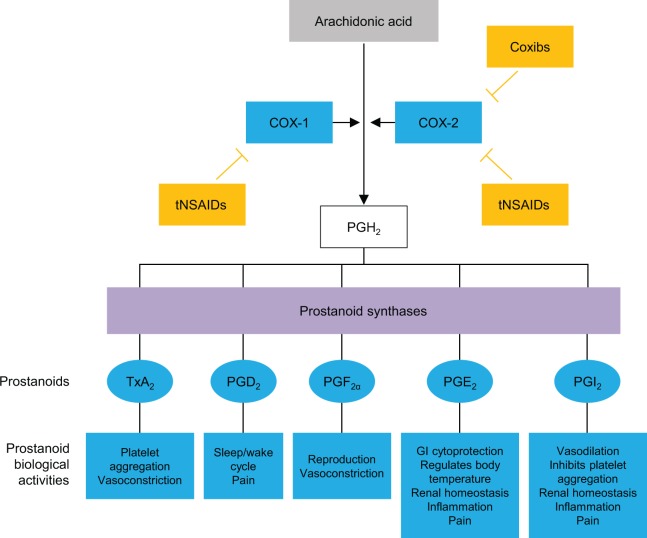
Mechanisms of action of traditional and COX-2-selective NSAIDs. Adapted from Brune and Patrignani.^37^ COX indicates cyclooxygenase; Coxibs, COX-2 inhibitors; GI, gastrointestinal; NSAIDs, nonsteroidal anti-inflammatory drugs; PG, prostaglandin; PGI_2_, prostacyclin; tNSAIDs, traditional NSAIDs; TxA_2_, thromboxane.

Whereas COX-1 inhibition is associated with GI and hematologic side effects, selective inhibition of COX-2 has been associated with CV side effects.^38^ Traditional, nonselective NSAIDs decrease the synthesis of prostaglandins via inhibition of both COX-1 and COX-2, while COX-2-selective NSAIDs primarily inhibit COX-2 (Figure 1).^37^ Acetaminophen decreases the synthesis of prostaglandins by acting as a substrate of the peroxidase cycles of COX-1 and COX-2.^48^ Acetaminophen also has endocannabinoid system effects.^49^

Mechanistically, the CV risk from nonselective NSAIDs is believed to be caused by profound inhibition of PGI_2_, which increases platelet reactivity, in conjunction with incomplete and intermittent inhibition of platelet COX-1. In this scenario, higher ratios of COX-2 to COX-1 inhibition appear to confer greater risk of thrombotic CV events.^50^ Additionally, competitive binding by NSAIDs at the COX-1 binding site of ASA on the platelet surface may lead to interference with the cardioprotective effects of ASA.^51^ Nonsteroidal anti-inflammatory drugs may also have direct or indirect effects on blood pressure (BP),^52,53^ including reductions in sodium excretion due to inhibition of prostaglandin E_2_ and interference with antihypertensive drugs, particularly angiotensin-converting enzyme inhibitors and angiotensin receptor blockers.^4^ Confounding factors during NSAID use include the fact that inflammatory conditions are associated with CV complications and that some CV medications may have less efficacy or altered pharmacokinetics in inflammatory states.^54,55^

## Pathophysiologic Effects of NSAIDs on BP

Prostaglandin E_2_ decreases renal Na^+^ reabsorption at the cortical thick ascending limb of the loop of Henle.^41^ Thus, NSAIDs, via COX inhibition, lead to increased Na^+^ reabsorption and an increase in plasma volume. However, in individuals with normal kidney function who are not taking antihypertensive therapies, mechanisms of homeostasis cause the nephrons to adapt within days of starting NSAIDs and sodium excretion returns to baseline. In contrast, homeostatic mechanisms may be impaired in older persons, those with chronic kidney disease, and people on diuretics and renin–angiotensin blocking agents, and sodium excretion never recuperates.^4^ This creates the potential for weight gain, edema, and congestive heart failure. The NSAIDs may also affect potassium (K^+^) homeostasis.^41^ Prostacyclin stimulates renin release and ultimately leads to an increase in K^+^ secretion by the distal nephron; its inhibition by NSAID treatment may lead to a type 4 renal tubular acidosis and hyperkalemia. Prostacyclin, an important vasodilator, is synthesized by the kidneys to maintain renal perfusion when a decrease in actual or effective circulating volume occurs. Nonsteroidal anti-inflammatory drugs administration in this setting may decrease renal blood flow substantially, leading to an acute kidney injury syndrome. Importantly, the risk of acute kidney injury associated with NSAIDs may be related to dose and duration of treatment, as well as volume status at the time of initiation of the NSAID.^41^

## Effects of OTC Analgesics in Normotensive and Hypertensive Persons

### Normotensive Participants

Furey and colleagues^56^ conducted a meta-analysis of 15 single-dose, randomized, double-blind trials evaluating the safety of OTC ibuprofen (200 or 400 mg; n = 878) and APAP (650 or 1000 mg; n = 849) versus placebo (n = 852) in healthy participants with acute pain conditions. The AE frequency did not differ significantly among groups (2.4% randomized to ibuprofen, 3.2% with APAP, and 2.1% with placebo), and no renal AEs were reported.^56^ DeArmond et al^57^ conducted a meta-analysis of 48 studies (27 single-dose studies and 21 multiple-dose studies) that assessed OTC naproxen safety in healthy participants with acute pain conditions; the analysis included studies with ibuprofen arms (n = 19 studies), APAP arms (n = 9 studies), or placebo arms. In total, 4138 participants received naproxen (187.5-400 mg naproxen or 220-440 mg naproxen sodium), 1574 participants received ibuprofen (200 or 400 mg), 671 participants received APAP (500-1000 mg), and 2423 participants received placebo. Rates of AEs were low and similar among all active treatment groups.^57^ Rainsford and colleagues^27^ evaluated the safety of OTC ibuprofen and APAP from 96 randomized, double-blind studies. No differences in AE rates were noted in any system organ class in participants receiving OTC ibuprofen or APAP for <7 days.^27^ In a meta-analysis of 8 randomized, double-blind, placebo-controlled trials that compared OTC ibuprofen (800-1200 mg/d; n = 1094) with placebo (n = 1093), significantly fewer (*P* = .018) participants receiving ibuprofen (300 [27.4%]) experienced an AE compared with those receiving placebo (346 [31.7%]).^58^

### Patients With Hypertension

Furey and coworkers^59^ investigated the renal effects of ibuprofen 400 mg 3 times a day compared with APAP 650 mg 3 times a day and ASA 650 mg 3 times a day for 7 days in a randomized, double-blind study of 25 patients aged ≥60 years with hydrochlorothiazide-treated stage 1 hypertension and mild renal insufficiency (serum creatinine 1.3-3.0 mg/dL). No significant changes from baseline in supine diastolic BP (DBP) occurred with ibuprofen or APAP; small decreases in DBP with ASA were not clinically significant. No significant changes in supine systolic BP (SBP) occurred with any of the OTC analgesics tested. Furthermore, no significant changes from baseline occurred for creatinine clearance, serum creatinine, potassium, blood urea nitrogen, or sodium in any treatment arm.^59^

Houston and colleagues^60^ studied 162 patients aged 18 to 75 years with stage 1 and 2 hypertension (DBP 90-115 mm Hg) stabilized to an average sitting DBP of <90 mm Hg on the calcium channel blocker verapamil. Participants were randomized to OTC ibuprofen (400 mg 3 times a day), naproxen (250 mg twice a day), or placebo for 3 weeks; BP was measured on days 7 and 21 of treatment. No significant differences from placebo were observed with ibuprofen or naproxen for seated SBP or DBP. Mean end-of-treatment change in DBP (the primary efficacy end point) adjusted for center and baseline BP was 1.7, 2.5, and 2.1 mm Hg for the ibuprofen, naproxen, and placebo groups, respectively. Similar percentages of participants in all groups had ≥10 mm Hg increases in either SBP (17%, 16%, and 15%, respectively) or DBP (2%, 9%, and 9%, respectively).^60^

The risk of CV events is increased with long-term use of NSAIDs and COX-2 inhibitors in patients with coronary artery disease^61,62^; thus, APAP is commonly used for analgesia in such patients. Sudano and coworkers evaluated the effect of APAP on BP in a randomized, double-blind crossover study of 33 patients aged 18 to 80 years who had coronary artery disease and were stabilized on CV medication for at least 1 month.^63^ Participants received APAP (1 g 3 times a day) and placebo for 2 weeks, separated by a 2-week washout period between treatments. Acetaminophen was associated with a significant increase in SBP (from 122.4 [11.9] to 125.3 [12.0] mm Hg; *P* = .021) compared with placebo (from 122.7 [11.6] to 122.2 [10.5] mm Hg). In addition, DBP increased significantly with APAP treatment (from 73.2 [6.9] to 75.4 [7.9] mm Hg; *P* = .024) compared with placebo (from 74.4 [6.9] to 74.6 [7.2] mm Hg). No effect on endothelial function, endothelial progenitor cells, or platelet adhesion was noted. Due to the short-term nature of this study, CV events were not observed.^63^

## Cardiovascular Events From Clinical Trials

There are no single randomized, controlled trials that compared OTC use of ibuprofen, naproxen, or APAP adequate to assess CV risk with low doses of these medications. Results of the PRECISION trial have shown that even with administration of prescription NSAID doses to participants with increased CV risk, a large number of participants treated over a long duration was needed to elicit a number of events sufficient to attempt to separate treatments from each other. Indeed, the primary outcome of death due to CV causes including hemorrhagic death occurred in 2.3%, 2.5%, and 2.7% of celecoxib-, naproxen-, and ibuprofen-treated patients, respectively, over a mean follow-up period of 34 [13] months.^26^ Furthermore, a prespecified analysis for those remaining on drug (on-treatment analysis) in PRECISION showed even lower rates of the primary CV end point in all 3 treatment groups (1.7%, 1.8%, and 1.9% on celecoxib, naproxen, and ibuprofen, respectively). However, there are numerous epidemiological studies that have investigated specific CV risks associated with total daily doses of analgesics that are in the range of OTC dosing, albeit with generally much longer treatment durations than are recommended with OTC use. This research is discussed below.

## Contrasting OTC and Prescription Doses of NSAIDs on CV Outcomes From Observational Databases

Andersohn and colleagues^29^ conducted a nested case–control study in a cohort of 486 378 participants aged ≥40 years registered in the UK General Practice Research Database (GPRD) who had at least 1 prescription for an NSAID between June 1, 2000, and October 31, 2004. They matched 3643 cases of MI from this group with 13 918 controls based on age, sex, general practice, and year of cohort entry. Mean follow-up duration was 542 days in both cohorts. At ibuprofen doses ≤1200 mg/d, the risk of MI was 0.99 (95% CI: 0.81-1.21); at doses >1200 mg/d, it was 1.14 (95% CI: 0.74-1.77). At naproxen doses ≤750 mg/d, the risk of MI was 1.19 (95% CI: 0.79-1.80); at doses >750 mg/d, it was 1.05 (95% CI: 0.66-1.66). Risk of MI was not significant for either ibuprofen or naproxen across the dose ranges studied.^29^

The influence of NSAIDs and APAP on the risk of major CV events (nonfatal MI, fatal coronary heart disease, and nonfatal and fatal stroke) was examined in a prospective cohort study of 70 971 women without known CV disease or cancer who participated in NHS I.^64^ Over 12 year follow-up, 2041 major CV events were confirmed. Women who reported occasional use of NSAIDs or APAP (defined as 1-21 days per month) did not experience a significantly increased CV risk compared with nonusers. After adjustment for CV risk factors and other analgesic classes, women who frequently used NSAIDs (≥22 days per month) had a relative risk (RR) of a CV event of 1.44 (95% CI: 1.27-1.65) compared with nonusers. Those who frequently used APAP (≥22 days per month) had an adjusted odds ratio (OR) of 1.35 (95% CI: 1.14-1.59). The elevated risk associated with frequent NSAID use was particularly evident among current smokers (RR: 1.82; 95% CI: 1.38-2.42; *P*_interaction_ = .02) and was absent among those who never smoked; smoking did not influence the risk with APAP use. Significant dose–response relationships were evident: Compared with nonusers, the RR for a CV event among women who used ≥15 tablets per week was 1.86 (95% CI: 1.27-2.73; *P*_trend_ ≤.001) for NSAIDs and 1.68 (95% CI: 1.10-2.58; *P*_trend_ = .002) for APAP.^64^

A population-based retrospective cohort study in participants aged 50 to 84 years between January 2000 and October 2005 with a nested case–control analysis (n = 8852 cases; n = 20 000 controls) of data from The Health Improvement Network UK database examined the effect of dose on the risk of nonfatal MI associated with current NSAID use.^30^ The RR of MI with OTC ibuprofen doses up to 1200 mg/d was not increased over that with nonuse (RR: 1.00; 95% CI: 0.80-1.25). For ibuprofen doses >1200 mg/d (most studies in this group assessed 1800 mg/d), the RR of MI was increased slightly, but not significantly so. The RR of MI with OTC naproxen doses up to 750 mg/d, 0.90 (95% CI: 0.50-1.60), was not increased over that with nonuse nor was it increased at higher prescription-strength doses (>750 mg/d, RR: 1.12; 95% CI: 0.74-1.69).^30^

A retrospective cohort study using UK GPRD data from 1987 to 2006 investigated the risk of MI with NSAID use in participants aged ≥40 years.^31^ In those with a first prescription for ibuprofen (8.2% of whom were prescribed doses >1200 mg/d), the relative rate of MI during ibuprofen use overall versus controls was 1.04 (95% CI: 0.98-1.09), indicating no increased risk. The relative rate of MI increased with ibuprofen dose. With ibuprofen dosing of 1200 mg/d, 1201 to 2399 mg/d, and ≥2400 mg/d, the relative rates (95% CIs) of MI were 1.02 (95% CI: 0.94-1.11), 1.22 (95% CI: 1.03-1.44), and 1.96 (95% CI: 1.05-3.65), respectively. Those with a first prescription for naproxen (1.9% of whom were prescribed doses >1000 mg/d) had a relative rate of MI during naproxen use overall of 1.03 (95% CI: 0.94-1.13), indicating no increased risk with naproxen. Higher doses of naproxen were not associated with increased relative rates of MI (RR [95% CI]: <1000 mg/d, 0.99 [0.85-1.17]; 1000 mg/d, 1.12 [0.98-1.27]; >1000 mg/d, 0.92 [0.49-1.71]). When exposure (dose) differences were taken into account, MI risk was comparable in current and past long-term users of NSAIDs. Ibuprofen, diclofenac, and naproxen were associated with similar absolute MI risk with similar histories of NSAID use. Linear trends for cumulative or daily dose revealed no statistical differences between ibuprofen, diclofenac, and ibuprofen.^31^

A nested case–control study from the Netherlands examined the risk of first hospitalization for MI or other CV events (unstable angina, cerebrovascular accident, transient ischemic attack [TIA]) in COX-2 and traditional NSAID users in the PHARMO Record Linkage System between January 1, 2001, and December 31, 2004.^32^ Users were classified as remote (users whose drug supply ended >60 days prior to the event date), recent (users with prescriptions that ended between 1 and 60 days prior to the event date), or current users (users whose duration of prescription overlapped with event date). The adjusted OR for MI risk with celecoxib use was 3.04 (95% CI: 1.31-7.04) for current high-dose use (ie, >1 defined daily dose) and 1.41 (95% CI: 0.62-3.17) for current low-dose use (ie, ≤1 defined daily dose) versus remote use. The adjusted OR for MI risk with current high-dose (>1200 mg/d) ibuprofen use was 1.66 (95% CI: 0.92-3.00) and 1.51 (95% CI: 1.06-2.14) with current low-dose (≤1200 mg/d) ibuprofen use versus remote use. Participants using any dose of ibuprofen had a significantly elevated OR for the risk of first hospitalization for MI (OR: 1.56; 95% CI: 1.19-2.05), but not for the risk of first hospitalization due to other CV events (OR: 1.18; 95% CI: 0.98-1.44). Respective ORs for first hospitalization for MI or any CV event were not significant with naproxen (MI, OR: 1.21 [95% CI: 0.87-1.68]; CV event, OR: 1.18 [95% CI: 0.94-1.47]).^32^

National administrative registers in Denmark were used to assess the risk of death and MI with NSAID use in 1 028 437 apparently healthy individuals aged ≥10 years who did not have a claim for selected concomitant pharmacotherapy from after 1995 to the date of their first claimed NSAID prescription or June 1, 2001.^33^ The HR for death with ibuprofen doses ≤1200 mg/d was 0.78 (95% CI: 0.73-0.84; *P* < .01), and the HR for the MI and death composite end point was 0.92 (95% CI: 0.86-0.97; *P* < .01). In contrast, the HR with ibuprofen doses >1200 mg/d was significantly higher for both death (HR: 1.77; 95% CI: 1.55-2.02; *P* < .01) and the MI and death composite end point (HR: 1.84; 95% CI: 1.62-2.08; *P* < .01). The HR for death was significantly lower at naproxen doses ≤500 mg/d (HR, 0.70; 95% CI: 0.58-0.86; *P* < .01), and the HR for the MI and death composite end point was 0.90 (95% CI: 0.76-1.06). At naproxen doses >500 mg/d, the HR was not significantly higher for death (HR: 1.25; 95% CI: 0.90-1.72) or for the MI and death composite end point (HR: 1.28; 95% CI: 0.95-1.74).^33^

Using this same Danish population of healthy individuals (N = 1 028 437) followed between January 1, 1997, and December 31, 2005, Fosbol and colleagues^34^ analyzed the risk of coronary death or nonfatal MI and stroke according to NSAID dose. Any use of ibuprofen (median dose 1200 mg/d) was associated with an increased OR for coronary death or nonfatal MI (OR: 1.52; 95% CI: 1.25-1.85; *P* < .01) and fatal or nonfatal stroke (OR: 1.29; 95% CI: 1.02-1.63; *P* < .05) compared with no use. At ibuprofen doses ≤1200 mg/d, the risk of coronary death or nonfatal MI was significantly increased (OR: 1.45; 95% CI: 1.19-1.77; *P <* .01), but the risk of fatal or nonfatal stroke was not (OR: 1.21; 95% CI: 0.95-1.53). At ibuprofen doses >1200 mg/d, the OR was not significantly increased for coronary death or nonfatal MI (OR: 1.44; 95% CI: 0.91-2.27) but was significantly increased for fatal or nonfatal stroke (OR: 1.36; 95% CI: 0.84-2.19; *P* < .05). Any use of naproxen (median dose 500 mg/d) was associated with an increased OR for fatal or nonfatal stroke (OR: 1.91; 95% CI: 1.04-3.50; *P* < .05) but not for coronary death or nonfatal MI compared with no use. There was no evidence of increased risk of coronary death, nonfatal MI, or fatal/nonfatal stroke with naproxen according to dose.^34^

McGettigan and Henry^35^ compared the risk of serious CV events among participants using COX-2-selective NSAIDs or nonselective NSAIDs in a systematic review and meta-analysis of 23 studies (17 case–control analyses and 6 cohort analyses). No significantly increased risk of serious CV events was identified for celecoxib (summary RR: 1.06; 95% CI: 0.91-1.23), ibuprofen (summary RR: 1.07; 95% CI: 0.97-1.18), naproxen (summary RR: 0.97; 95% CI: 0.87-1.07), piroxicam (summary RR: 1.06; 95% CI: 0.70-1.59), or meloxicam (summary RR: 1.25; 95% CI: 1.00-1.55). Diclofenac (summary RR: 1.40; 95% CI: 1.16-1.70) and indomethacin (summary RR: 1.30; 95% CI: 1.07-1.60) were associated with increases in risk of serious CV events.^35^

In a 2011 update of this work,^36^ McGettigan and Henry expanded their review (30 case–control and 21 cohort studies) to include new drugs and also attempted to look at OTC use risk by assessing CV event risk with low NSAID doses of short duration in populations at low risk. Of the drugs evaluated in 10 or more studies, the highest overall RRs for major CV events were observed with rofecoxib (pooled RR: 1.45; 95% CI: 1.33-1.59) and diclofenac (pooled RR: 1.40; 95% CI: 1.27-1.55) and the lowest risks were observed for ibuprofen (pooled RR: 1.18; 95% CI: 1.11-1.25) and naproxen (pooled RR: 1.09; 95% CI: 1.02-1.16). When low versus high doses of the available OTC treatments (ibuprofen, naproxen, and diclofenac) were compared, only diclofenac was associated with a statistically significantly increased risk of CV events (RR: 1.22; 95% CI: 1.12-1.33) at low doses (variably defined as <100 mg/d, ≤100 mg/d, or <150 mg/d). In studies where low-dose ibuprofen (variably defined as ≤1200 mg/d, ≤1600 mg/d, or <1800 mg/d, depending on the study) or low-dose naproxen (variably defined as ≤500 mg/d, ≤750 mg/d, or ≤1000 mg/d) were studied, neither ibuprofen (RR: 1.05; 95% CI: 0.96-1.15) nor naproxen (RR: 0.97; 95% CI: 0.87-1.08) was associated with an increased risk of CV events. At high doses, there was a significantly increased risk of major CV events with ibuprofen (RR: 1.78; 95% CI: 1.35-2.34), but not with naproxen (RR: 1.05; 95% CI: 0.89-1.24).^36^

Most recently, Bally and colleagues^65^ performed an individual patient data meta-analysis of studies derived from health-care databases to examine the time course for the risk of MI, as well as the effects of dose and use duration for commonly used NSAIDs. The cohort of 446 763 individuals included 61 460 cases of MI and 385 303 controls. Overall, results from the analysis suggested that: (i) the current use of NSAIDs was associated with a significantly increased risk of MI; (ii) the risk onset for MI was rapid, occurring in the first week of usage; (iii) a longer treatment duration does not seem to be associated with a higher probability of increased MI risk; and (iv) the risk of MI decreases over time since the last time of NSAID use.^65^ Interestingly, findings (ii) and (iii) are not consistent with most other epidemiological studies reviewed here, nor are they consistent with findings from controlled clinical trials in which risk increases over time.^26^ It is possible that this reflects protopathic bias, in which patients are prescribed NSAIDs due to pain and/or inflammatory conditions, both of which can increase the risk of CV events.

In addition, there were differences in MI risk that were related to dose and duration of treatment. Any current dose of ibuprofen taken for 1 to 7 days was associated with an adjusted OR (95% CI) of 1.48 (1.00-2.26) for the risk of MI. When ibuprofen was taken at a daily dose of ≤1200 mg/d for 8 to 30 days or for >30 days, the adjusted ORs (95% CIs) for the risk of MI were 1.04 (0.72-1.35) and 1.32 (1.02-1.74), respectively. At ibuprofen doses >1200 mg/d taken for 8 to 30 days or for >30 days, the adjusted ORs were 1.75 (1.00-2.93) and 1.47 (1.04-2.04), respectively, for the risk of MI. For naproxen, any current dose taken for 1 to 7 days was associated with an adjusted OR (95% CI) of 1.53 (1.07-2.33) for the risk of MI. When naproxen was taken at a daily dose of ≤750 mg/d for 8 to 30 days or for >30 days, the adjusted ORs for risk of MI were 1.23 (0.90-1.61) and 1.21 (0.95-1.52), respectively. At naproxen doses >750 mg/d taken for 8 to 30 days or for >30 days, the adjusted ORs for the risk of MI were 1.76 (1.14-2.65) and 1.21 (0.91-1.57), respectively.^65^ A summary of observational study findings is presented in Table 2. It should be noted that the majority of these studies do not demonstrate an increased risk of CV events with low NSAID doses.

**Table 2.** Risk of CV Events According to Daily Dose or Dose Frequency of Current NSAID or APAP Use.

Reference	PubMed ID Number	Sample Size	Study Type	Main Outcome of Interest	Low-Dose (or Low-Frequency) Use	Risk (95% CI)	High-Dose (or High-Frequency) Use	Risk (95% CI)
Andersohn et al, 2006^29^	16618816	486 378 persons ≥40 years of age registered within the UK GPRD (June 01, 2000, to October 31, 2004); 3643 cases of MI; 13 918 controls (matched for age, sex, practice, and year of cohort entry)	Nested case–control study; 542 days (SD: 390.2) of follow-up	Risk of MI	IBU ≤1200 mg/d	MRR: 0.99 (0.81-1.21)	IBU >1200 mg/d	1.14 (0.74-1.77)
					NAP ≤750 mg/d	MRR: 1.19 (0.79-1.80)	NAP >750 mg/d	1.05 (0.66-1.66)
Chan et al, 2006^64^	16534006	70 971 women 44-69 years of age (1990-2002); 2041 major CV events^a^	Prospective cohort study; 12 years of follow-up	Risk of MI	1-21 days/month use vs nonuse	No significant risk of CV events with NSAID or APAP use: MRR^b^ for NSAIDs: 1-4 days: 0.95 (0.79-1.14); 5-14 days: 1.00 (0.81-1.22); 15-21 days: 0.91 (0.67-1.23)	≥22 days/month use vs nonuse	MRR^b^ for NSAIDs: 1.44 (1.27-1.65)
								MRR^b^ for APAP: 1.35 (1.14-1.59)
						MRR^b^ for APAP: 1-4 days: 0.98 (0.84-1.14); 5-14 days: 1.09 (0.91-1.30); 15-21 days: 1.22 (0.95-1.56)		
Garcia Rodriguez et al, 2008^30^	18992652	716 395 persons 50-84 years of age from the THIN database (January 2000 to October 2005); 8852 nonfatal MI cases vs 20 000 controls	Population-based, retrospective, nested case–control study; average of 4.1 years of follow-up	Risk of MI	IBU ≤1200 mg/d	RR^c^ for IBU vs nonuse: 1.00 (0.80-1.25)	IBU >1200 mg/d (mainly 1800 mg/d)	RR^c^ for IBU vs nonuse: 1.56 (0.90-2.71)
					NAP ≤750 mg/d	RR^c^ for NAP: 0.90 (0.50-1.60)	NAP >750 mg/d	RR^c^ for NAP: 1.12 (0.74-1.69)
van Staa et al, 2008^31^	18624902	729 294 NSAID users and 443 047 disease-matched controls from UK GPRD (1987-2006)	Retrospective cohort study; mean follow-up in NSAID users and matched controls: 6.1 and 5.6 years, respectively	Risk of MI	IBU <1200 mg/d	RR^d^: 1.05 (0.91-1.22)	IBU 1201-2399 mg/d	RR^d^: 1.22 (1.03-1.44)
					IBU 1200 mg/d	RR^d^: 1.02 (0.94-1.11)		
							IBU ≥2400 mg/d	RR^d^: 1.96 (1.05-3.65)
					NAP <1000 mg/d	RR^d^: 0.99 (0.85-1.17)	NAP 1000 mg/d NAP >1000 mg/d	RR^d^: 1.12 (0.98-1.27) RR^d^: 0.92 (0.49-1.71)
van der Linden et al, 2009^32^	18495734	485 059 NSAID users (remote, recent, or current) from the PHARMO Record Linkage System (January 01, 2001 to December 31, 2004); 2196 and 5500 cases of first hospitalization for MI or CV event, respectively	Nested case–control study within a historical cohort	Risk of first hospitalization for MI or other CV events (unstable angina, CVA, TIA)	IBU ≤1 DDD IBU, defined as ≤1200 mg/d	OR for MI (low-dose IBU vs remote use): 1.51 (1.06-2.14)	IBU >1 DDD (IBU >1200 mg/d)	OR for MI (high-dose IBU vs remote use): 1.66 (0.92-3.00)
Fosbol et al, 2009^33^	18987620	1 028 437 apparently healthy individuals ≥10 years of age from the Danish Register of Medicinal Product Statistics (1997-2005)	Historical cohort study design	CV risk (death and MI) with NSAID use	IBU ≤1200 mg/d	HR for death: 0.78 (0.73-0.84; *P* < .01); HR for composite end point^e^: 0.92 (0.86-0.97; *P* < .01)	IBU >1200 mg/d	HR for death: 1.77 (1.55-2.02; *P* < .01); HR for composite end point: 1.84 (1.62-2.08; *P* < .01)
					NAP ≤500 mg/d	HR for death: 0.70 (0.58-0.86; *P* < .01); HR for composite end point: 0.90 (0.76-1.06)	NAP >500 mg/d	HR for death: 1.25 (0.90-1.72); HR for composite end point: 1.28 (0.95-1.74)
Fosbol et al, 2010^34^	20530789	1 028 437 apparently healthy individuals ≥10 years of age from the Danish Register of Medicinal Product Statistics (January 01, 1997 to December 31, 2005)	Historical cohort study design	Risk of coronary death or nonfatal MI or stroke	IBU ≤1200 mg/d vs no use	OR for coronary death or nonfatal MI: 1.45 (1.19-1.77; *P* < .01); OR for fatal/nonfatal stroke: 1.21 (0.95-1.53)	IBU >1200 mg/d vs no use	OR for coronary death or nonfatal MI: 1.44 (0.91-2.27); OR for fatal/nonfatal stroke: 1.36 (0.84-2.19; *P* < .05)
					NAP ≤500 mg/d vs no use	OR for coronary death or nonfatal MI: 1.37 (0.83-2.27); OR for fatal/nonfatal stroke: 1.52 (0.81-2.87)	NAP >500 mg/d vs no use	OR for coronary death or nonfatal MI: 0.24 (0.06-1.03); OR for fatal/nonfatal stroke: 2.50 (0.57-10.96)
McGettigan and Henry, 2006^35^	16968831	NR	Systematic review of case–control (n = 17) or cohort design (n = 6) studies reporting on CV risks with use of COX-2 inhibitors and conventional NSAIDs	Risk of serious CV events	IBU: all doses	Summary RR: 1.07 (0.97-1.18)	IBU: all doses	Summary RR: 1.07 (0.97-1.18)
					NAP: all doses	Summary RR: 0.97 (0.87-1.07)	NAP: all doses	Summary RR: 0.97 (0.87-1.07)
McGettigan and Henry, 2011^36^	21980265	NR	Systematic review of case–control (n = 30) or cohort design (n = 21) studies reporting on CV risks with use of COX-2 inhibitors and conventional NSAIDs	Assessment of the risk of major CV events associated with individual NSAIDs at different doses	IBU: variably defined as ≤1200 mg/d (n = 8 studies), ≤1600 mg/d (n = 1 study), or <1800 mg/d (n = 2 studies)	RR for CV events: 1.05 (0.96-1.15)	IBU: variably defined as >1200 mg/d (n = 8 studies), >1600 mg/d (n = 1 study), or ≥1800 mg/d (n = 2 studies)	RR for CV events: 1.78 (1.35-2.34)
					NAP: variably defined as ≤500 mg/d (n = 2 studies), ≤750 mg/d (n = 4 studies), or ≤1000 mg/d (n = 4 studies)	RR for CV events: 0.97 (0.87-1.08)	NAP: variably defined as >500 mg/d (n = 2 studies), >750 mg/d (n = 4 studies), or >1000 mg/d (n = 4 studies)	RR for CV events: 1.05 (0.89-1.24)
Bally et al, 2017^65^	28487435	446 763 individuals (61 460 cases of MI vs 385 303 controls) derived from health-care database studies	Systematic review	Risk of MI	IBU ≤1200 mg/d, 8-30 days	Adjusted OR: 1.04 (0.72-1.35)	IBU >1200 mg/d, 8-30 days	1.75 (1.00-2.93)
					IBU ≤1200 mg/d, >30 days	Adjusted OR: 1.32 (1.02-1.74)	IBU >1200 mg/d, >30 days	1.47 (1.04-2.04)
					NAP ≤750 mg/d, 8-30 days NAP ≤750 mg/d, >30 days	Adjusted OR: 1.23 (0.90-1.61) Adjusted OR: 1.21 (0.95-1.52)	NAP >750 mg/d, 8-30 days NAP >750 mg/d, >30 days	1.76 (1.14-2.65) 1.21 (0.91-1.57)

Abbreviations: APAP, acetaminophen; BMI, body mass index; CI, confidence interval; COPD, chronic obstructive pulmonary disease; COX-2, cyclooxygenase-2; CV, cardiovascular; CVA, cerebrovascular accident; DDD, defined daily dose; DM, diabetes mellitus; GP, general practitioner; HR, hazard ratio; HTN, hypertension; IBU, ibuprofen; IRR, incidence rate ratio; MI, myocardial infarction; MRR, multivariate relative risk; NAP, naproxen; NR, not reported; NSAID, nonsteroidal anti-inflammatory drug; OR, odds ratio; RA, rheumatoid arthritis; RR, relative risk or relative rate; SD, standard deviation; SLE, systemic lupus erythematosus; THIN, The Health Improvement Network; TIA, transient ischemic attack; UK GPRD, UK General Practice Research Database.

^a^Nonfatal MI, nonfatal stroke, fatal coronary event, fatal stroke.

^b^Multivariate relative risk adjusted for age; parental history of MI before age 60 (yes/no); history of DM (yes/no); history of hypercholesterolemia (yes/no); smoking history; history of HTN (yes/no); BMI; regular, moderate, or vigorous exercise; postmenopausal hormone use, current multivitamin use (yes/no); and energy-adjusted quintiles of folate, omega-3 fatty acids, saturated fat, alcohol, and other analgesic categories.

^c^Relative risk adjusted for age, sex, calendar year, BMI, GP visits, referrals, smoking, Townsend score, ischemic heart disease, DM, RA, COPD, and anticoagulants, antihypertensives, oral steroids, and aspirin use.

^d^Adjusted for age; gender; calendar year smoking history; use of alcohol; BMI; socioeconomic class of practice location; region of practice; number of visits to the GP 6 to 12 months before; history of DM, HTN, systemic inflammation (RA or SLE), ischemic heart disease, cerebrovascular disease, and renal failure; and prescribing in the 6 months before use of diuretics, statins, oral glucocorticoids, aspirin, anticoagulants, and cardiac glycosides prior to the index date.

^e^Composite end point = MI or death.

## Coadministration of NSAIDs With ASA

Aspirin and NSAIDs share a common binding site on the COX-1 enzyme^66,67^; consequently, there is the potential for a competitive and meaningful interaction between these therapies on platelet function. Results of a 6-day crossover study with single daily doses of analgesics (ibuprofen, APAP, rofecoxib, ASA) and a 6-day parallel-group study with multiple daily doses of analgesics (ibuprofen, diclofenac, ASA) showed that administration of 400 mg ibuprofen 2 hours before (single daily-dose crossover study) or 2, 7, and 12 hours after administration of ASA (parallel-group study with multiple daily doses) interfered with the thromboxane inhibition normally observed after ASA administration, suggesting that ibuprofen might limit the cardioprotective effect of ASA.^51^ In contrast, the pharmacodynamics of ASA were not affected by concomitant administration of rofecoxib, APAP, or diclofenac.^51^

In a post hoc analysis of the Physicians’ Health Study, a 5-year randomized, double-blind, placebo-controlled trial of administration of 325 mg ASA every other day (with observational data on NSAID use), Kurth and colleagues^68^ investigated NSAID inhibition of the clinical benefit of ASA 325 mg every other day on first MI among 22 071 healthy US physicians aged 40 to 84 years. Risk of MI was not increased with intermittent NSAID use (1-59 days/year) in either the ASA group or the placebo group; however, NSAID use for ≥60 days/year was associated with a significant increase in MI in the ASA group. Among those randomized to ASA, the multivariable-adjusted RR of MI was 1.21 (95% CI: 0.78-1.87) and 2.86 (95% CI: 1.25-6.56) for NSAID use on 1 to 59 days/year or on ≥60 days/year, respectively, compared with no use. Among those randomized to placebo, the RR of MI was 1.14 (95% CI: 0.81-1.60) and 0.21 (95% CI: 0.03-1.48) for NSAID use on 1 to 59 days/year and for NSAID use on ≥60 days/year, respectively, compared with no prior use. Thus, individuals who used ASA 325 mg every other day and used NSAIDs on ≥60 days per year had a >2-fold increased risk of MI, while intermittent NSAID use had no apparent effect on the cardioprotective effects of ASA.^68^

MacDonald and Wei^69^ studied participants in the United Kingdom who were discharged from the hospital with a CV diagnosis (MI, angina, stroke, or TIA) and peripheral vascular disease who used ASA (<325 mg/d) and who survived at least 1 month after discharge. Participants were divided into 4 groups: (1) ASA alone, (2) ASA plus ibuprofen, (3) ASA plus diclofenac, and (4) ASA plus any other NSAIDs. Of the 7107 participants included (age range, 27-100 years), 6285 received ASA alone, 187 received ASA plus ibuprofen, 206 received ASA plus diclofenac, and 429 received ASA plus other NSAIDs. During a median follow-up of 3.3 years (interquartile range, 1.7-5.5), 2266 (32%) participants died. The mean (SD) dose of ibuprofen was 1210 (384.4) mg/d and of diclofenac was 117 (33.5) mg/d. A significantly higher risk of all-cause mortality (HR: 1.93; 95% CI: 1.30-2.87; *P* = .0011) and CV mortality (HR: 1.73; 95% CI: 1.05-2.84; *P* = .0305) was noted in the ASA plus ibuprofen group versus the group using ASA alone; in contrast, diclofenac was not associated with an interaction with ASA that increased CV risk. Although this study showed that coadministration of ibuprofen with low-dose ASA in participants with known CV disease significantly increased the risk of all-cause and CV mortality,^69^ it is limited by a lack of adjustment for either frequency or actual dose of NSAID.

Cryer and colleagues^70^ conducted a prospective, double-blind, randomized, placebo-controlled study in which 51 participants received chewable, immediate-release ASA (81 mg once daily) for 8 days and were then randomized to receive ASA followed at ∼1, 7, and 13 hours by either ibuprofen 400 mg 3 times a day (the maximum labeled OTC dose) or placebo for 10 days. Results showed no evidence of loss of the cardioprotective effect of ASA with ibuprofen. Thromboxane B_2_ inhibition in the ibuprofen group was >90% on all days tested in all participants. These data suggest that if participants are already on chronic ASA, ibuprofen does not have a deleterious effect.^70^

In 2006, the FDA advised both health-care professionals and consumers of the potential interaction between ibuprofen and low-dose ASA that might render ASA less effective in terms of its antiplatelet effect.^71^ The FDA advised that the interaction is minimized if ibuprofen is given ≥8 hours before or ≥30 minutes after immediate-release ASA. In addition, the FDA stated that the risk of attenuation of the antiplatelet effect of low-dose ASA is likely to be minimal with “occasional” use of ibuprofen.^71^ In contrast, because of the risk of bleeding with more potent antiplatelet drugs (eg, clopidogrel, ticagrelor) or the novel oral anticoagulants, NSAIDs should not be used concomitantly with these agents.

## Conclusions and Perspectives

**Figure 2.**
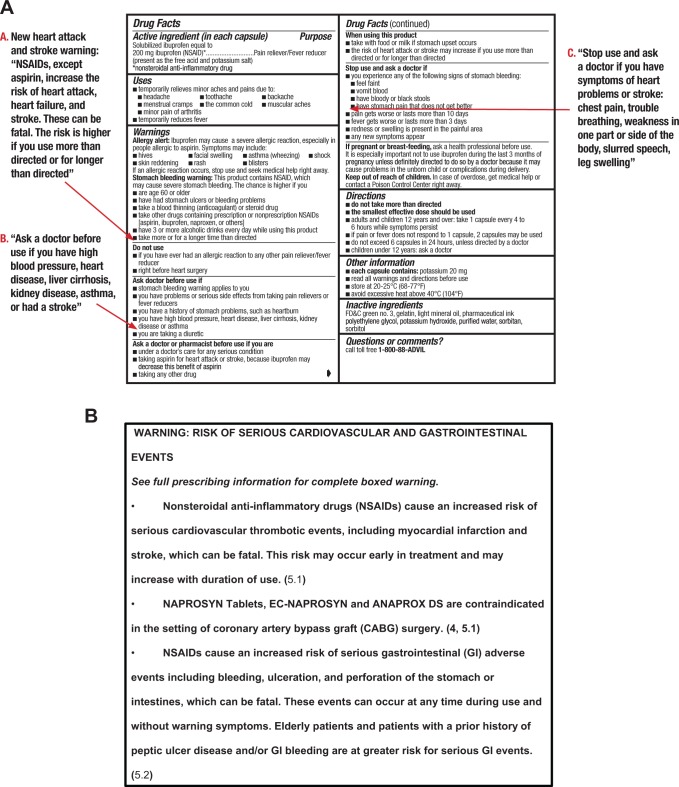
Warning labels for OTC (A) or prescription (B) NSAIDs. A, Over-the-counter NSAIDs^72^: All OTC NSAID Drug Facts Labels are being revised for US FDA approval. New drug safety information will be added to the respective sections as shown. B, Black box warning for prescription-strength naproxen. All prescription NSAIDs carry the same warning.^12^ FDA indicates Food and Drug Administration; NSAIDs, nonsteroidal anti-inflammatory drugs; OTC, over-the-counter.

Based on a comprehensive review of CV safety data on prescription NSAIDs, the warning labels of OTC NSAIDs have been updated recently to emphasize their potential CV risks and to reinforce the importance of following OTC dosing instructions. A comparison of NSAID warnings for OTC versus prescription products is noted in Figure 2.^12,72^ These warnings are consolidated in the labels to make them more easily understood and to discourage overuse of OTC NSAIDs. In addition, we must keep in mind that both NSAIDs and APAP are also present in combination products that are available OTC, such as combinations with antihistamines for sleep aids, increasing the overall exposure to these agents, and it is important to query patients about these agents when conducting a thorough medication use history. Our review demonstrates that CV safety data from studies using prescription-strength analgesic doses cannot be extrapolated to assess OTC safety, because prescription doses and durations of use are far greater than the recommended duration of OTC NSAID use (typically <10 days). Available data from randomized, controlled trials of analgesics at OTC doses are also insufficient to assess CV safety because data are typically derived through extraction of “OTC” data from prescription databases with durations of use that are much longer than specified in OTC product labels. Data from these sources do demonstrate lower rates of CV events with OTC doses compared with the higher dose prescription doses, particularly for ibuprofen. Results of PRECISION demonstrate much lower than expected absolute CV event rates with prescription-strength NSAIDs in an arthritis population enriched for CV disease. Hence, while the absolute CV risk of OTC NSAIDs is not evident from clinical trials, observational data support low risk, particularly with short-term exposure. As with any treatment, individual patient characteristics should be taken into account before specific recommendations for OTC analgesic use are provided.
